# Corpus creation and language identification for code-mixed Indonesian-Javanese-English Tweets

**DOI:** 10.7717/peerj-cs.1312

**Published:** 2023-06-22

**Authors:** Ahmad Fathan Hidayatullah, Rosyzie Anna Apong, Daphne T.C. Lai, Atika Qazi

**Affiliations:** 1 School of Digital Science, Universiti Brunei Darussalam, Bandar Seri Begawan, Brunei; 2 Department of Informatics, Universitas Islam Indonesia, Sleman, Yogyakarta, Indonesia; 3 Centre for Lifelong Learning, Universiti Brunei Darussalam, Bandar Seri Begawan, Brunei

**Keywords:** Code-mixing, Language identification, Indonesian, Javanese, English, Twitter, BERT

## Abstract

With the massive use of social media today, mixing between languages in social media text is prevalent. In linguistics, the phenomenon of mixing languages is known as code-mixing. The prevalence of code-mixing exposes various concerns and challenges in natural language processing (NLP), including language identification (LID) tasks. This study presents a word-level language identification model for code-mixed Indonesian, Javanese, and English tweets. First, we introduce a code-mixed corpus for Indonesian-Javanese-English language identification (IJELID). To ensure reliable dataset annotation, we provide full details of the data collection and annotation standards construction procedures. Some challenges encountered during corpus creation are also discussed in this paper. Then, we investigate several strategies for developing code-mixed language identification models, such as fine-tuning BERT, BLSTM-based, and CRF. Our results show that fine-tuned IndoBERTweet models can identify languages better than the other techniques. This is the result of BERT’s ability to understand each word’s context from the given text sequence. Finally, we show that sub-word language representation in BERT models can provide a reliable model for identifying languages in code-mixed texts.

## Introduction

Today, mixing languages is prevalent in daily communication, especially in informal situations, such as texting posts on social media. In linguistics, combining two or more languages within an utterance of speech or text is called code-mixing (Hoffmann, 2014; Ritchie & Bhatia, 2012). Mixing languages is particularly common in regions where people are natively multilingual.

Indonesia is one of the world’s most multilingual countries, with over 700 local spoken languages (Aji et al., 2022). More than 198 million and 84 million people speak Indonesian and Javanese, respectively (Eberhard, Simons & Fennig, 2021). Hence, mixing Indonesian and Javanese in an utterance is common in Indonesia, especially among the Javanese people. Besides, exposure to English from social media and school makes Indonesians mix their languages with English (Rizal & Stymne, 2020). As a result, mixing Indonesian, Javanese, and English in daily conversation becomes the most prevalent language combination in Indonesian societyt (Yulianti et al., 2021).

The following is an example of a code-mixed sentence containing Indonesian, Javanese, and English words:

*“Aku udah coba ngedownload tapi error, tulung aku diewangi downloadke panduane!”*

(*English:* ***I have tried to download but error, please help me download the guideline!***).

The sentence contains the following language compositions: Indonesian *(aku, udah, coba, tapi),* mix Indonesian-English *(ngedownload)*, English *(error)*, Javanese *(tulung, aku, diewangi)*, mix Javanese-English *(downloadke)*, and mix Indonesian-Javanese *(panduane)*. In the above sentence, the mixing of languages occurs not only within the sentence but also within the word. For example, the word *‘ngedownload’* consists of *‘nge-‘* (informal Indonesian prefix) and *‘download’* (English). The word *‘downloadke’* consists of *‘download’* (English) and *‘-ke’* (Javanese suffix). The word *‘panduane’* consists of *‘panduan’* (Indonesian) and *‘-e’* (Javanese suffix).

To analyze code-mixed text, a language identification (LID) task is often used as part of the pre-processing step (Hidayatullah et al., 2022). LID is critical for some subsequent natural language processing tasks in code-mixed documents (Gundapu & Mamidi, 2018). Applying LID in the code-mixed text has become a foundation work of various NLP systems, including sentiment analysis (Ansari & Govilkar, 2018; Mahata, Das & Bandyopadhyay, 2021), translation (Barik, Mahendra & Adriani, 2019; Mahata et al., 2019), and emotion classification (Yulianti et al., 2021). The absence of LID in pre-processing tasks can affect those NLP systems. For example, if the language is not accurately identified, a code-mixed sentence will produce an inaccurate translation. In another case, an offensive content identification system may produce incorrect results if the words in a sentence are not correctly identified (Singh, Sen & Kumaraguru, 2018).

However, most existing NLP systems are designed to process a single language at once (Sabty et al., 2021). The number of NLP systems that can process multiple languages per sentential unit is restricted (Nguyen et al., 2021). The traditional language identification systems fail to detect languages correctly from mixed language texts (Kalita & Saharia, 2018). Processing multiple languages within a sentence requires additional processing tasks compared to monolingual texts due to various language combinations such as sentence, clause, word, and sub-word levels (Mave, Maharjan & Solorio, 2018). Detecting language from code-mixed text using a traditional approach like dictionary lookup is no longer applicable. The dictionary approach produces poor results due to spelling inconsistencies and the loss of word context (Ansari & Govilkar, 2018).

On the other hand, the availability of annotated code-mixed data, including Indonesian and Javanese data, remains limited. Even though Indonesian and Javanese have many speakers, only a few studies have addressed the code-mixing phenomenon in the Indonesian language (Adilazuarda et al., 2022; Winata et al., 2022). In comparison to the languages spoken in Europe, the existence of Indonesian and Javanese languages in NLP research is relatively understudied (Aji et al., 2022).

Considering the problems above, this study makes the following contributions:

1. We develop an annotated code-mixed corpus for Indonesian-Javanese-English language identification (IJELID) from Twitter data.
2. To build the language identification model, we apply a fine-tuning strategy based on pre-trained Bidirectional Encoder Representations from Transformers (BERT) models introduced by Devlin et al. (2019). BERT has shown outstanding performance when fine-tuned for any downstream NLP tasks (Ulčar & Robnik-Šikonja, 2020). We also seek to use several strategies using CRF and BLSTM-based models.

## Related Work

### Code-mixed data availability for language identification

In this study, we collect some papers focused on conducting language identification for code-mixed text. As a result, we found 17 related studies published between 2016 and 2022. During that period year, we identify 14 code-mixed datasets such as Manipuri-English (Lamabam & Chakma, 2016), Konkani-English (Phadte & Wagh, 2017), Telugu-English (Gundapu & Mamidi, 2018), Bengali-English (Jamatia, Das & Gambäck, 2018; Mandal & Singh, 2018), Hindi-English (Ansari et al., 2021; Jamatia, Das & Gambäck, 2018; Mandal & Singh, 2018; Shekhar, Sharma & Beg, 2020), Bengali-Hindi-English (Jamatia, Das & Gambäck, 2018), Turkish-English (Yirmibeşoğlu & Eryiğit, 2018), Indonesian-English (Barik, Mahendra & Adriani, 2019; Yulianti et al., 2021), Sinhala-English (Smith & Thayasivam, 2019), Arabic-English (Sabty et al., 2021), English-Assamese-Hindi-Bengali (Sarma, Singh & Goswami, 2022), Telugu-English (Kusampudi, Chaluvadi & Mamidi, 2021), Malayalam-English (Thara & Poornachandran, 2021), and Kannada-English (Shashirekha et al., 2022; Tonja et al., 2022).

From those 14 datasets, five studies utilized Indonesian-related code-mixed datasets. Barik, Mahendra & Adriani (2019) introduced code-mixed Indonesian-English data from Twitter for the text normalization task. Yulianti et al. (2021) used the dataset created by Barik, Mahendra & Adriani (2019) to see the impact of code-mixed normalization on the emotion classification task. In Yulianti et al. (2021), they introduced new feature sets to improve the performance of the language identification task. Suciati & Budi (2019) created a review dataset containing mixed Indonesian-English. They gathered the review data from a culinary website for an aspect-based opinion mining task. Arianto & Budi (2020) also developed a code-mixed Indonesian-English dataset for aspect-based sentiment analysis. The dataset was collected from Google Maps reviews. Tho et al. (2021) proposed a code-mixed Indonesian-Javanese corpus using the Twitter dataset for sentiment analysis.

Among the five previous studies, two papers (Barik, Mahendra & Adriani, 2019; Yulianti et al., 2021) applied language identification in their research. The remaining studies focused on opinion mining and sentiment analysis. All five studies above provided bilingual code-mixed languages, namely Indonesian-English and Indonesian-Javanese. There have been no studies focusing on trilingual code-mixed language data, particularly code-mixed Indonesian-Javanese-English. The existing studies do not concentrate on the language identification task. Instead, they aimed to solve problems in sentiment analysis, emotion classification, and translation. Since no dataset is available for code-mixed Indonesian-Javanese-English, it is necessary to develop a dataset for such mixed languages. The availability of a code-mixed Indonesian-Javanese-English dataset can help improve NLP research for related Indonesian languages.

### Language identification for code-mixed text

Many studies have developed language identification models for code-mixed text using various techniques. CRF has been utilized in many research due to its simplicity and impressive performance in building a code-mixed language identification model (Gundapu & Mamidi, 2018). Lamabam & Chakma (2016) applied a trigram-based model using J48 and CRF for code-mixed English-Manipuri data. Their experiments revealed that the CRF with the first three and last three-character features gave the best result with an F1 score of 90%. Phadte & Wagh (2017) applied support vector machine (SVM), random forest, and CRF to build a word-level language identification system code-mixed Konkani-English text. In their study, CRF outperformed the other methods with an accuracy of 97%. Gundapu & Mamidi (2018) worked on English-Telugu code-mixed data by comparing four algorithms, naïve Bayes, random forest, hidden Markov model, and CRF. CRF achieved the best performance with an F1 score of 91% and an accuracy of 91.28%. Yirmibeşoğlu & Eryiğit (2018) presented a word-level language identification system for code-mixed Turkish-English using CRF and n-gram character level as a feature. The CRF could give a promising performance with an F1 score macro of 94.5%. Smith & Thayasivam (2019) built language identification models for code-mixed Sinhala-English. In their study, CRF outperformed SVM, LSTM, KNN, and random forest, with an overall F1 score of 94%. A study by Barik, Mahendra & Adriani (2019) conducted language identification using mixed Indonesian-English data from Twitter using CRF and obtained an F1 score of 89.58%. Yulianti et al. (2021) used the same dataset as Barik, Mahendra & Adriani (2019) to develop a language identification system. They applied various techniques, such as CRF, CRF++, CNN, CNN+CRF, and RNN. The results showed that CRF++ performed the best, with an F1 score of 93.98%.

Mandal & Singh (2018) proposed a multichannel neural network-based method for code-mixed Bengali-English and Hindi-English. The multichannel module combines convolutional neural network (CNN) and long-short term memory (LSTM). In addition, they added a bidirectional-LSTM-CRF module to capture the context of the input text. Jamatia, Das & Gambäck (2019) presented a Hindi-Bengali-English code-mixed language identification using LSTM and BLSTM. Based on their experiments, the BLSTM performed better than the LSTM, with an F1 score of 87.07%. Shekhar, Sharma & Beg (2020) built a code-mixed language identification model for Hindi-English using Bidirectional LSTM (BLSTM). Their study revealed that BLSTM achieved an F1 score of 93.97%.

Sabty et al. (2021) utilized a character BLSTM and segmental recurrent neural network (SegRNN) for the Arabic-English text language identification system. The SegRNN achieved the best results in their experiments with an F1 score of 94.84%. Sarma, Singh & Goswami (2022) studied a word-level language identification system for Assamese, Bengali, Hindi, and English code-mixed data utilizing CNN and BLSTM with pre-trained word embeddings. Their experiments indicated that the CNN performed better than the BLSTM, with an F1 score of 90.79%. Kusampudi, Chaluvadi & Mamidi (2021) created a code-mixed Telugu-English corpus. They conducted word-level language identification using BLSTM-CRF with an accuracy of 99.32%.

Thara & Poornachandran (2021) developed a large-scale Malayalam-English dataset for code-mixed language identification using BERT-based models, such as XLM-RoBERTa, ELECTRA, CamemBERT, and DistilBERT. Compared to the other BERT models, ELECTRA obtained the highest F1 score of 99.33% and an accuracy of 99.41%. Ansari et al. (2021) leveraged BERT base and RoBERTa models for code-mixed Hindi-English language identification. Their experiments showed that pre-training and fine-tuning using code-mixed Hindi-English-Urdu text yielded an F1-score of 84%, higher than the pre-trained monolingual models. Shashirekha et al. (2022) developed a code-mixed Kannada-English dataset called CoLI-Kenglish. In their study, three different approaches were applied, such as machine learning-based (CoLI-vectors and CoLI-ngram), deep learning-based (CoLI-BiLSTM), and transfer learning-based (CoLI-BiLSTM). CoLI-ngrams using morphological features outperformed all other models with an average macro F1-score of 64%. Tonja et al. (2022) combined LSTM and BERT for code-mixed Kannada-English language identification. Their proposed model gained a weighted F1 score of 84% and a macro F1 score of 61%.

To summarize, NLP researchers have applied various techniques for code-mixed language identification tasks. CRF has shown a satisfying performance among traditional machine learning techniques. LSTM-based architectures have demonstrated promising results in several studies. The emergence of the transformer (Vaswani et al., 2017) has improved NLP research. Furthermore, transformer-based methods, such as BERT and its variants, have become a breakthrough and state-of-the-art in solving NLP problems (Wolf et al., 2020). BERT-based models have shown remarkable performance in the code-mixed language identification task and outperformed traditional neural network techniques, such as RNN and CNN (Thara & Poornachandran, 2021).

## Corpus Creation

### Data collection and pre-processing

We collect 15K tweets in several batches from December 2021 to July 2022. In this work, we list some code-mixed Indonesian, Javanese, and English keywords to obtain the tweets to ensure that the retrieved tweets are code-mixed. In the pre-processing tasks, we first filter the tweets by removing duplicates. Subsequently, we replace user mentions, URLs, and hashtags with @user, httpurl, and #hashtag. Finally, we convert all words into lowercase. In our dataset, we notice some tweets containing languages other than Indonesian, Javanese, and English, such as Malay, Sundanese, Arabic, and Korean. The occurrence of Malay and Sundanese in the retrieved tweets is reasonable since both languages have similarities to Indonesian and Javanese. In addition, Arabic and Korean scripts exist in our dataset because people sometimes add both scripts to their tweets. In this case, we keep those languages in our dataset.

### Annotation guidelines

To develop the dataset, we employ two annotators who understand Indonesian, Javanese, and English. We cover standard and non-standard language labeling forms in the annotation process. Since we work on social media text, the characteristics of the data are different from the standard text. The texts consist of many informal forms, such as slang words, non-standard spellings, mixed letters with numbers, abbreviated words, and lengthening words. Hence, this study proposes an annotation guideline to cope with those informal texts. The following is an annotation guideline to produce a standard annotation result:

1. This study introduces seven labels to annotate tokens from the dataset, namely: ID (Indonesian), JV (Javanese), EN (English), MIX_ID_EN (mixed Indonesian-English), MIX_ID_JV (mixed Indonesian-Javanese), MIX_JV_EN (mixed Javanese-English), and OTH (Other).
2. The ID label is used to annotate Indonesian words, including both standard and non-standard forms of Indonesian words.
3. The EN label is used to annotate English words, including both standard and non-standard forms of English words.
4. The JV label is used to annotate Javanese words, including both standard and non-standard Javanese words.
5. The MIX_ID_EN label is used to annotate words containing mixed Indonesian-English. For example:
  • *diprint (printed)* → *di- (Indonesian prefix) + print (English).*
  • *filenya (the file)* →* file (English) + -nya (Indonesian suffix).*
6. The MIX_ID_JV label is used to annotate words containing mixed Indonesian-Javanese. For instance:
  • *bajune (the clothes)* →* baju (Indonesian) + ne (Javanese suffix).*
  • *penjuale (the seller)* →* penjual (Indonesian) + e (Javanese suffix).*
7. The MIX_JV_EN label is used to annotate words containing mixed Javanese-English. For example:
  • *endinge (the ending)* →* ending (English) + -e (Javanese suffix).*
  • *ngemailke (send email)* → *ng- (Javanese prefix) + email (English) + -ke (Javanese prefix).*
8. The OTH (Other) label is used to annotate the following:
  • symbols, punctuations, and number,
  • unknown words, words other than Indonesian, Javanese, and English,
  • Twitter entities: username or mention,
  • URL, emoji, emoticons, date, time, and currency,
  • laugh expressions *(haha, xixixi, wkwkwk)* and sad expressions *(huhuhu, hiks).*
9. If a word *(w)* exists in more than one language, it will be annotated as a particular language (ID, JV, or EN), depending on the context.
  • If *w* is preceded and followed by Javanese words, *w* is annotated as JV.
  • If *w* is preceded and followed by Indonesian words, *w* is annotated as ID.
  • If *w* is preceded and followed by English words, *w* is annotated as EN.
10. Foreign or borrowed words that exist in all languages are identified depending on the context of tweets or sentences.
11. A named entity followed by a possessive pronoun from a particular language can be labeled as ID or JV, depending on the context.
  • *Macbookku (My Macbook)* →* Macbook (named entity) + -ku (Indonesian or Javanese suffix).*
  • *Macbooknya (The Macbook)* →* Macbook (named entity) + -nya (Indonesian suffix).*

### Corpus creation challenges

This sub-section highlights some challenges encountered during the data creation process. Annotating language in Twitter data is not as simple as in formal text data due to the non-standard language forms. In this study, we define the non-standard language forms by categorizing into the following: mixed letters and numbers, slang, abbreviated words and acronyms, expressive lengthening, and English words written in Indonesian spelling style (Hidayatullah, 2015). Table 1 presents examples of non-standard form words in Indonesian, Javanese, and English.

**Table 1:** Examples of the non-standard form words in Twitter data.

**No**	**Type**	**Language**		
		**Indonesian**	**Javanese**	**English**
1	Mixed letters and numbers	•*‘se7’* stands for *‘setuju’* (to agree)	•*‘siji2’* stands for *‘siji-siji’* (one by one)	•*‘b4’* (before)
		•*‘anak2’* stands for *‘anak-anak’* (kids)		•*‘ni8t’* (night)
2	Slangs	•*‘santuy’* stands for *‘santai’* (relaxed)	•*‘ngunu’* stands for *‘ngono’* (like that)	•*‘epic’* (awesome)
		•*‘nobar’* stands for *‘nonton bareng’* (watch together)	•*‘bonek’* stands for *‘bondo nekat’* (reckless)	•*‘noob’* (newbie)
				•*‘menfess’* (mention confess)
				•*‘crashy’* (crazy and trashy)
3	Abbreviated words or acronym	•*‘dgn’* stands for *‘dengan’* (with)	•*‘kbh’* stands for *‘kabeh’* (all)	•*‘idk’ (I don’t know)*
		•*‘slkh’* stands for *‘sekolah’* (school)	•*‘mtrnwn’* stands for *‘matur nuwun’* (thank you)	•*‘dm’ (direct message)*
		•*‘SD’* stands for *‘sekolah dasar’* (elementary school)	•*‘lur’* stands for *‘sedulur’* (brother or sister)	•*‘omg’ (oh my god)*
				•*‘thx’ (thanks)*
				•*‘ur’* (your)
4	Expressive lengthening	•*‘senaaang’* stands for *‘senang’* (happy)	•*‘kaaabeeeh’* stands for *‘kabeh’* (all)	•*‘gooooood’* (good)
5	English words written in Indonesian spelling style	–	–	•‘*n’* (and)
				•*‘plis’* (please)
				•*‘wiken’* (weekend)
				•*donlod* (download)
				•*‘gud’* (good)
				•*‘selow’* (slow)

Another challenge in the annotation stage is determining words recognized in more than one language. For instance,

(1) *Guys, pertandingan* ***final*** *nanti malam jam berapa? Thanks*
***English***: *Guys, what time is the final match tonight? Thanks*
(2) *Finally, Liverpool menang di babak quarter* ***final*** *UCL*.
***English***: *Finally, Liverpool won in the UCL quarter-finals*.

From the two examples above, the word *‘final’* exists in Indonesian and English languages. Therefore, we must look at the surrounding words and the sentence’s whole context to determine the language label for the word *‘final’*. In the first example, the word *‘final’* is labeled as ID because the previous and the following words are Indonesian. In the second example, the word *‘final’* is labeled as EN because the term *‘quarter final’* is an English phrase.

Moreover, identifying intra-word code-mixing is also challenging due to the prefixes or suffixes that exist in the two languages. For example, the word *‘disave’* (saved) consists of *‘di-‘* (a prefix) followed by the English word *‘save’*. Another example is *‘smartphoneku’* (my smartphone), which contains *‘smartphone’* and the suffix *‘-ku’*. The prefix *‘di-‘* in the word *‘disave’* and the suffix *‘-ku’* in *‘smartphoneku’* exist in Indonesian and Javanese. Therefore, the word *‘disave’* and *‘smartphoneku’* can be annotated as MIX_ID_EN or MIX_JV_EN, depending on the context of the sentence. To determine the correct label, the annotators should consider the previous words, the following words, and the whole words of the sentence.

### Inter-annotator agreement

This study uses Cohen’s kappa (Cohen, 1960) to quantify the agreement between two annotators. The following is the equation for calculating Cohen’s kappa (*K*):

(1) \begin{eqnarray*}K= \frac{\mathit{Pr} \left( a \right) -Pr(e)}{1-Pr(e)} .\end{eqnarray*}

*Pr*(*a*) is the frequency with which two annotators assigned the same label. *Pr*(*a*) is obtained by calculating all agreed labels divided by the total data. *Pr*(*e*) is the probability of agreement when the annotators see the observed data randomly. The *Pr*(*e*) is calculated by summing the probability when both annotators randomly select the first label and the probability when both annotators select the second label. Cohen’s kappa value (*K*) spans from 1 to −1, indicating that annotators select different labels for each sample. A value of 0 indicates that the annotators agreed precisely as frequently as they would if they were both guessing randomly. Therefore, the closer the *K* value to 1, the better the dataset.

### Code-mixing index

After getting the distribution of each label from the dataset, we measure the complexity of code-mixing in the text using the code-mixing index (CMI) (Gambäck & Das, 2014). The CMI value is calculated using the following formula:

(2) \begin{eqnarray*}CMI= \left\{ \begin{array}{@{}l@{}} \displaystyle 100\times \left[ 1- \frac{\max \nolimits ({w}_{i:i=1,2,\ldots ,n})}{n-u} \right] , n\gt u \\ \displaystyle 0, n=u. \end{array} \right. \end{eqnarray*}

In Eq. (2), *w*_*i*_ is the number of words of the language *i*, max(*w*_*i*_) represents the number of words of the prominent language, *n* is the total number of tokens, and *u* represents the number of language independent tokens (such as named entities, abbreviations, mentions, and hashtags). For monolingual utterances, the CMI score equals to 0 (zero), since the $\max \left( {w}_{i} \right) =n-u$. A low CMI score implies monolingualism in the text, whereas a high CMI score indicates code-mixing.

## Language Identification Model

### Conditional random fields (CRF)

CRF is a discriminative model for sequential data labelling based on a conditional distribution approach (Lafferty, McCallum & Pereira, 2001). CRF employs contextual information from previous labels, thus increasing the amount of information for the model to generate an accurate label prediction. Let *X* be an input sentence containing sequence of words (*x*_1_, *x*_2_, *x*_3_, …, *x*_*n*_) and *Y* = (*y*_1_, *y*_2_, *y*_3_, …, *y*_*n*_) is the label prediction of such words. The CRF represents the likelihood of predicting the output *Y* given the input *X* as a conditional distribution *p*(*y*|*x*). The calculation of a linear-chain CRF is shown by Eq. (3) (Sutton & McCallum, 2012):

(3) \begin{eqnarray*}p \left( y{|}x \right) = \frac{1}{Z(x)} \prod _{t=1}^{T}\mathrm{exp} \left\{ \sum _{k=1}^{K}{\lambda }_{k}{f}_{k}({y}_{t},{y}_{t-1},{x}_{t}) \right\} .\end{eqnarray*}

In Eq. (3), *Z*(*x*) is a normalization factor function obtained using the following formula:

(4) \begin{eqnarray*}Z(x)=\sum _{y}\prod _{t=1}^{T}\mathrm{exp} \left\{ \sum _{k=1}^{K}{\lambda }_{k}{f}_{k}({y}_{t},{y}_{t-1},{x}_{t}) \right\} .\end{eqnarray*}

In Eqs. (3) and (4), $\lambda = \left\{ {\lambda }_{k} \right\} $ be a parameter vector weight estimated from the training set, and $F= \left\{ {f}_{k}({y}_{t},{y}_{t-1},{x}_{t}) \right\} {\scriptsize \begin{array}{@{}c@{}} \displaystyle K\\ \displaystyle k=1 \end{array}}$be a set of feature functions. *T* is the number of time step indexed by *t*.*K* is the number of features and *k* indexes the feature function *f*_*k*_ and weight *λ*_*k*_.

### BLSTM-based architecture

#### Input representation for BLSTM-based architecture

1. Word-level representation

**Figure 1:**
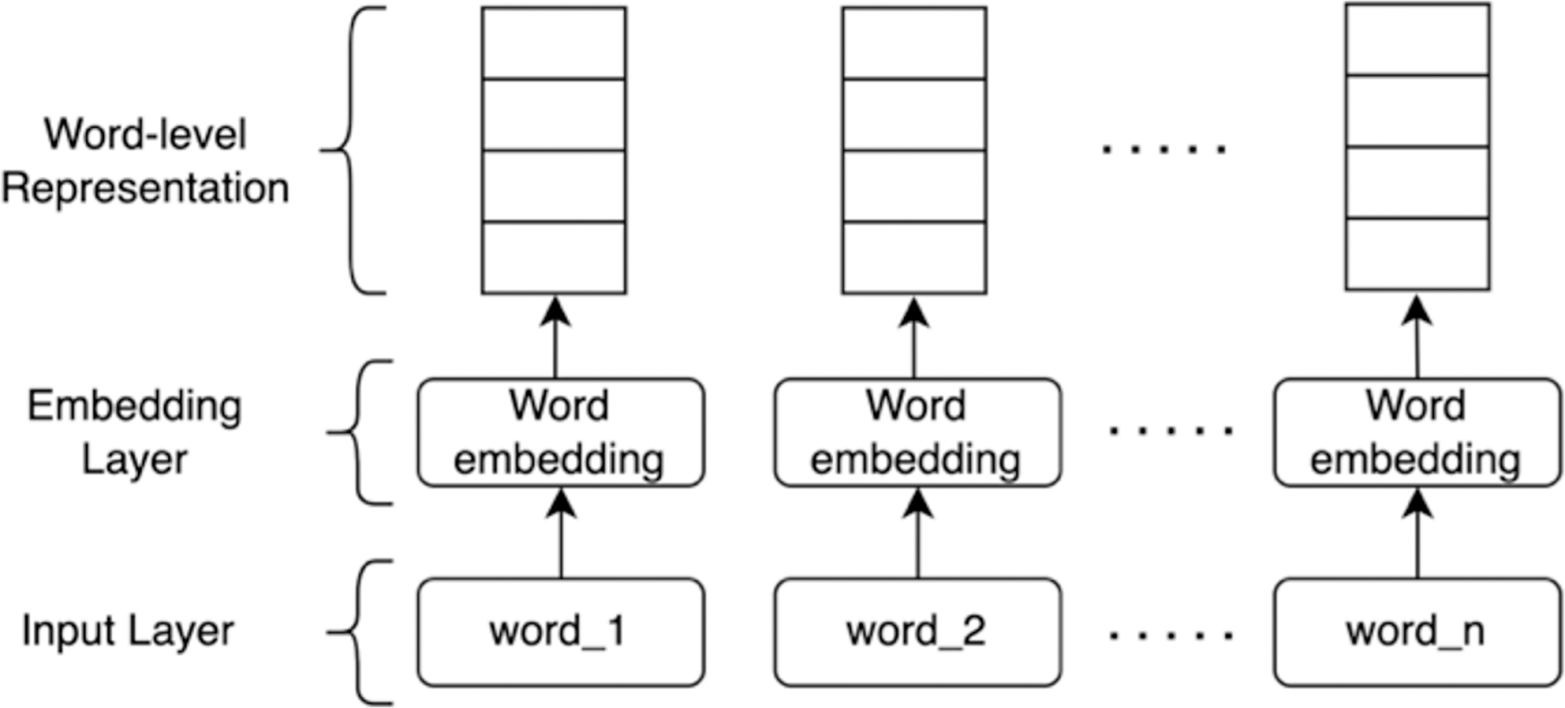
Word-level representation.

Word-level representation is a real-valued vector representing a word to capture the semantic relationship between words. Using word embedding, words with a closer meaning will have a similar vector representation. The embedding layer carries out the transformation from words to their corresponding vector representations. The embedding layer receives sentences in the form of a sequence of tokens. Each token is then transformed into a word vector with a fixed size by mapping the index of such a token. Figure 1 illustrates the word-level representation model.

2. Character-level representation

Character-level representation aims to capture morphological features of the words by processing the character that composes words (Mave, Maharjan & Solorio, 2018). Using character-level representation can help alleviate the out-of-vocabulary (OOV) problems in the text data (Joshi & Joshi, 2020). The results of character representation are used to augment the word vector representation before being processed through the classification layer.

**Figure 2:**
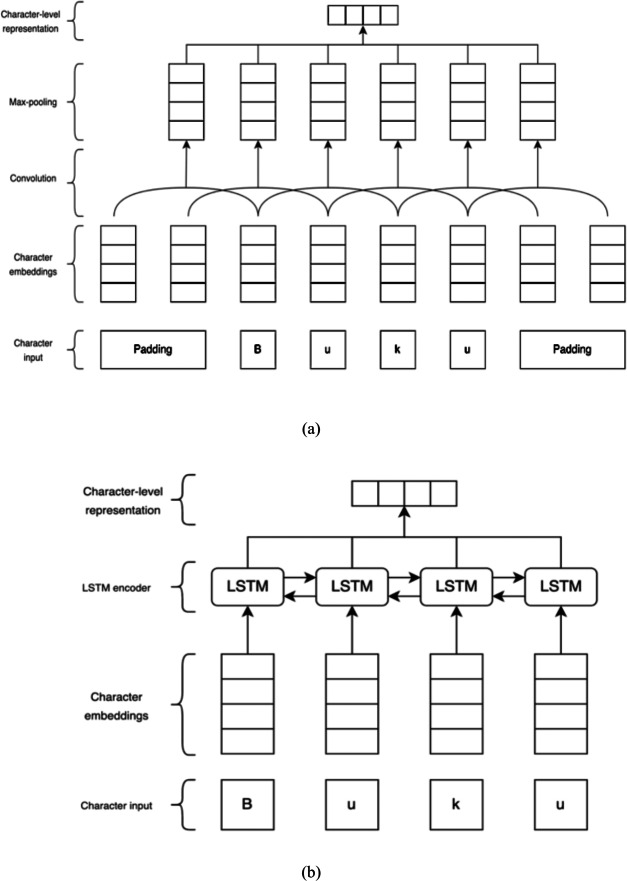
(A) Character CNN representation. (B) Character LSTM representation. These illustrations are adapted from Reimers & Gurevych (2017).

This study employs CNN and LSTM to train the character-level representation. In character-based CNN representation, a word is decomposed into a sequence of characters. The character inputs are passed into the character embedding layer. The embedding layer outputs are transmitted to the convolutional layer, which produces local features by applying a convolutional filter across a sliding n-character window. Subsequently, the max-pooling layer takes the maximum value over each dimension to represent a particular word. As for the character LSTM representation, we put a single LSTM layer on top of the embedding layer. The embedding outputs pass the character embedding vector via forward and backward LSTMs and combine each output to generate the encoding of the associated word. The character CNN and character LSTM representations are illustrated in Fig. 2.

#### Bidirectional long short-term memory (BLSTM)

BLSTM is an enhancement of LSTM networks that tries to increase model performance on sequence classification tasks (Hidayatullah, Cahyaningtyas & Pamungkas, 2020). By incorporating two distinct hidden layers, bidirectional LSTM (BLSTM) networks can access both preceding and subsequent contexts. These networks can efficiently capture long-distance relations in the sequence in both directions (Mave, Maharjan & Solorio, 2018). We initialize the embedding layer and feed the output sequence to the spatial dropout layer with a dropout. Subsequently, we add a single BLSTM layer and a recurrent sigmoid activation. BLSTM applies two LSTM networks by incorporating forward and backward hidden layers to capture previous and subsequent contexts (Liu & Guo, 2019). Lastly, a dense layer with a Softmax activation function is added to predict the final word label.

#### BLSTM-CRF

BLSTM-CRF is a combination of deep learning and traditional machine learning approaches. The combination between BLSTM and CRF has been proven to produce good results in sequence tagging tasks (Poostchi, Borzeshi & Piccardi, 2018). A CRF layer can be added to the top layer of the BLSTM architecture to predict the label of the entire phrase at the same time (Lafferty, McCallum & Pereira, 2001). In the sequence labeling task, it is critical to consider the association between neighboring labels. BLSTM, on the other hand, does not generalize the connection between output labels (Wintaka, Bijaksana & Asror, 2019). That is due to the probability distribution of BLSTM being independent. The combination of BLSTM and CRF may efficiently exploit past and future input features through an LSTM layer and sentence-level tag information through a CRF layer (Huang, Xu & Yu, 2015). In this BLSTM-CRF architecture, we stack the following layers: input, embedding, SpatialDropout1D, BLSTM, dense, and CRF.

#### BLSTM + character LSTM

In this architecture, we replicate the previous model proposed by Samih et al. (2016). This model receives two different inputs: word and character. Following that, the embedding layer processes the word and character inputs. A single LSTM layer is used to generate the character-level representations. These vector representations of characters are then concatenated with vector representations of words. The concatenated vector results then pass through SpatialDropout1D and a BLSTM layer with 100 hidden units. Finally, a dense layer is added to the BLSTM to predict the tag results.

#### BLSTM + character CNN

Similar to the BLSTM + character LSTM method, the input layer of this BLSTM + character CNN used word and character embeddings. In this architecture, the character-level representations are trained using CNN (Zhang, Zhao & LeCun, 2015). The character representations are obtained by passing the character input to a dropout layer followed by a Conv1D (kernel_size: 2, activation: ReLu) and a MaxPooling1D layer. The outputs are then flattened and fed into a dropout layer. A SpatialDropout1D layer receives the concatenated vectors between word and character representations. BLSTM proceeds with the output from the SpatialDropout1D and passes the results to the final dense layer to get the tag prediction.

### BERT-based architecture

#### BERT input representation

BERT has a particular set of rules for representing the input text, namely sub-word representation. Sub-word representation is an alternative solution between word and character-based representations. BERT uses the WordPiece tokenization algorithm to create the sub-word representation (Wu et al., 2016). WordPiece starts by establishing an initial vocabulary composed of elementary units and then increases this vocabulary to the desired size. The vocabulary begins with characters from a single language. Then the most common character combinations in the vocabulary are added iteratively. WordPiece learns merged rules for the character pairs and finds the pair that maximizes the likelihood of the training data. Equation (5) is the formula to calculate the score for each pair:

(5) \begin{eqnarray*}\mathrm{Score}= \frac{\text{frequency of pair}}{(\text{frequency of first unit}\times \text{frequency of second unit})} .\end{eqnarray*}

The score is calculated by dividing the frequency of the pair by the product of the frequencies of each of its components. The algorithm works by prioritizing the merging of pairs when each part occurs less frequently in the vocabulary. For example, the pair *‘read’* and *‘##ing’* will not be merged even though the token *‘reading’* frequently appears in the vocabulary. This is because the pair *‘read’* and *‘##ing’* will probably frequently occur in many other words. A pair between *‘re’* and *‘##ad’* will likely be merged since *‘re’* and *‘##ad’* appear less frequently individually. Therefore, the token *‘read’* is not split, while the token *‘reading’* is separated into *‘read’* and *‘ing’*. This teaches the idea that the token *‘reading’* is derived from *‘read’* with slightly different meanings but the same origin.

**Figure 3:**
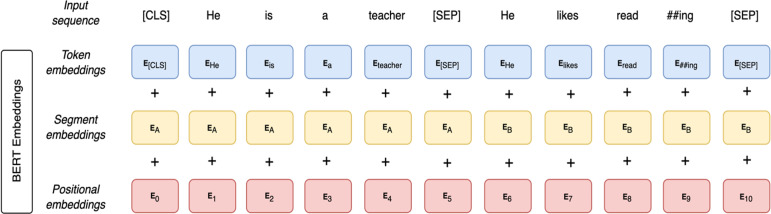
BERT input representation.

As illustrated in Fig. 3, BERT input representation consists of three embeddings: token embeddings, segment embeddings, and position embeddings. In the token embeddings, two special tokens are added to each sentence. At the beginning of each sentence, a [CLS] token is added. Another special token is a [SEP] token which is located at the end of each sentence. The [SEP] token is added to separate between sentences. It is used as a learned segment embedding denoting a token as part of segment A or B. Segment embeddings are sentence numbers encoded in a vector. The model identifies whether a specific token belongs to sentence A or B in the segment embeddings. Position embeddings provide information regarding the word order in the input sequence. Finally, the BERT representation is obtained by summing those three embeddings.

#### BERT

Bidirectional encoder representations from transformers (BERT) is a language representation model built using the transformer-based technique developed by Google (Devlin et al., 2019). BERT is a transformer encoder stack capable of simultaneously reading a whole sequence of inputs. The BERT architecture is a deep bidirectional model, meaning that BERT takes information from both the left and right sides of the token’s context during the training task. BERT employs a self-multi-headed transformer-based attention model pre-trained on a large multilingual corpus (Dowlagar & Mamidi, 2022). BERT pre-trains the raw text data in a self-supervised way, which means there is no human annotation to generate inputs and labels from those texts.

BERT is pre-trained with two objectives, masked language modeling (MLM) and next sentence prediction (NSP). MLM randomly masks 15% of the words in the input text, then runs the whole masked sentence through the model to predict the masked words. For the NSP task, the model concatenates two masked sentences during pre-training and occasionally matches sentences adjacent to each other in the original text. The model then predicts whether the two sentences follow each other.

#### Fine-tuning BERT

**Figure 4:**
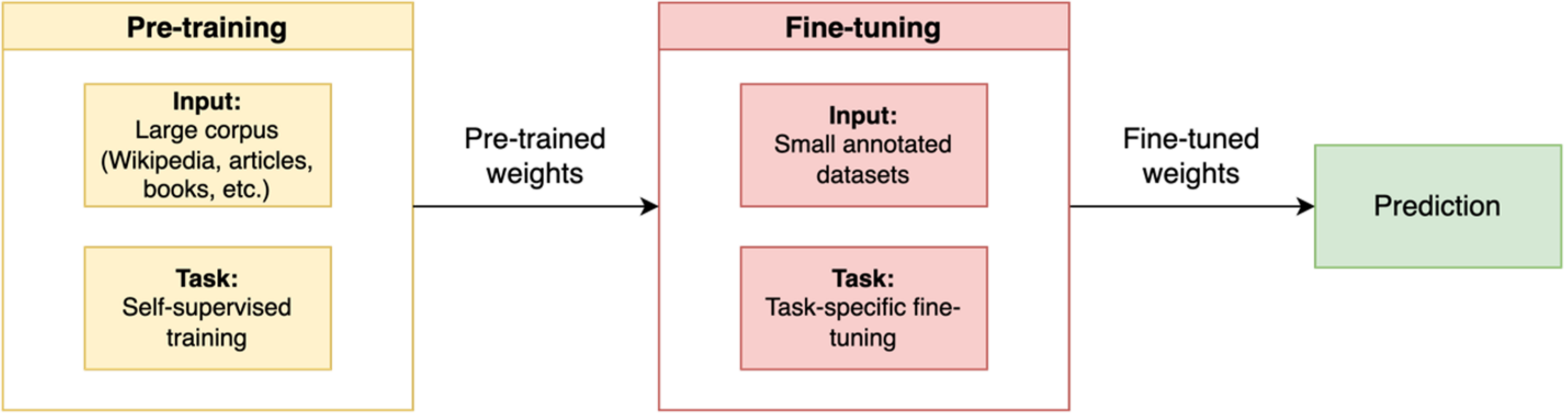
Pre-training and fine-tuning BERT.

As illustrated in Fig. 4, fine-tuning is done by leveraging a pre-trained model and then training it on a particular dataset suited to a specific task. The BERT model is first set with the pre-trained weight parameters. Next, all parameters are fine-tuned using annotated data from the downstream tasks. Ultimately, the fine-tuned weights are then used for the prediction task.

**Figure 5:**
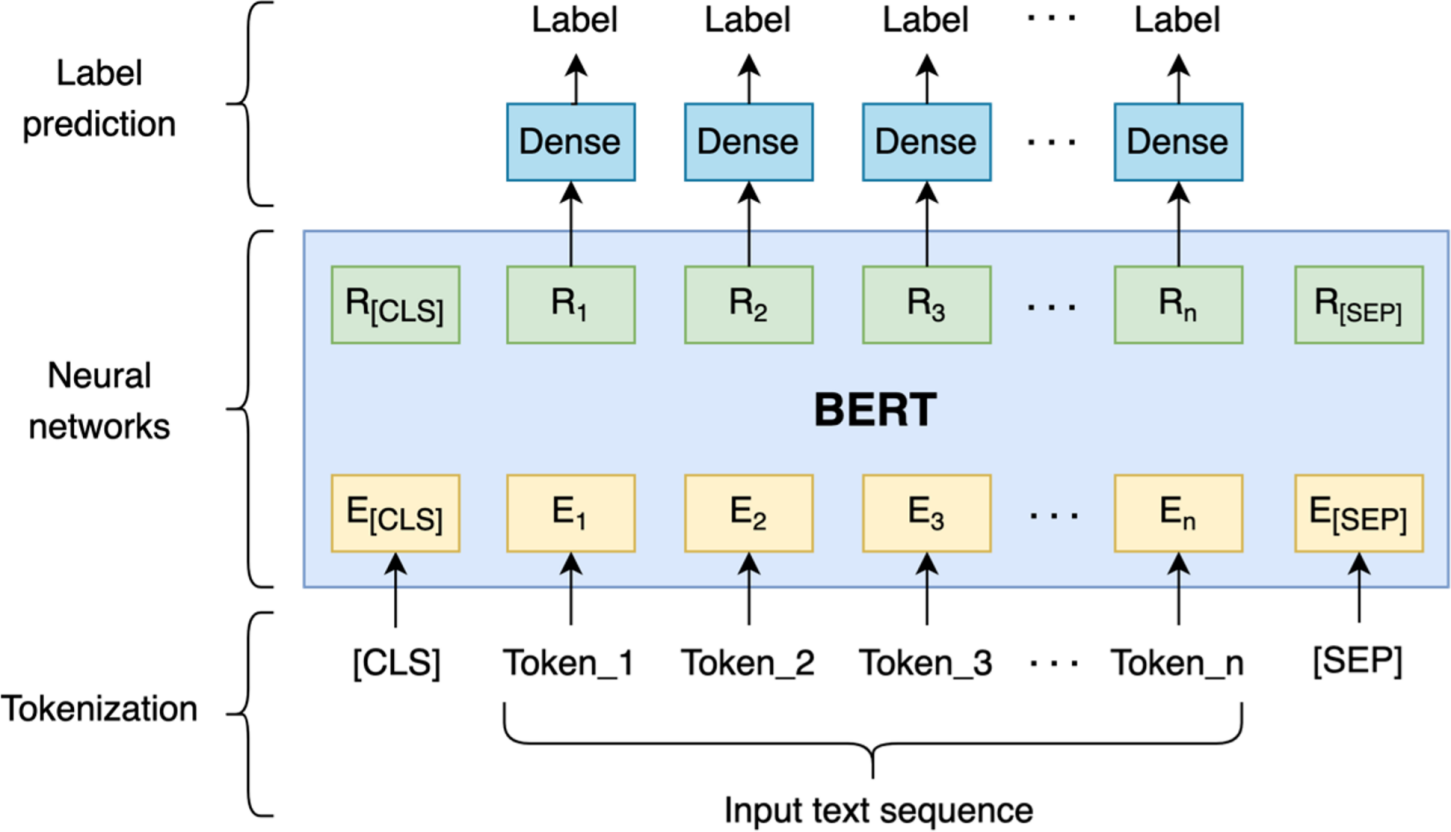
Fine-tuning BERT for code-mixed language identification.

In this study, the fine-tuning tasks are performed by leveraging two existing pre-trained BERT models, namely multilingual BERT (mBERT) (https://huggingface.co/bert-base-multilingual-cased) and IndoBERTweet (https://huggingface.co/indolem/indobertweet-base-uncased). The first pre-trained model is created based on the multilingual BERT. Multilingual BERT is a masked language modeling (MLM) objective-trained model (Devlin et al., 2019). It is trained with a large Wikipedia corpus on top of 104 languages, including English, Indonesian, and Javanese. Another pre-trained model used to build the language identification model is IndoBERTweet. IndoBERTweet is a pre-trained domain-specific model using a large set of Indonesian Twitter data (Koto, Lau & Baldwin, 2021).

The illustration of fine-tuning BERT for the language identification task can be seen in Fig. 5. The [CLS] symbol and [SEP] are added at the beginning and the end of a single text sequence. Each token of the sequence and the contextual representation of each token are denoted by *E* and *R*, respectively. Following that, the BERT representation of each token is fed into dense layers. In the dense layers, the dense layer parameters are shared to get the label of each token.

### Experimental setup

We conduct the experiments by splitting our dataset into training (6,170 tweets), validation (1,543 tweets), and testing (3,306 tweets). The training is performed using an 80 cores CPU, 250GB of RAM, and 4 GPUs (NVIDIA Tesla V100 SXM2). Before training, we apply hyperparameter tuning to get the optimal hyperparameters for each technique to maximize model performance.

For CRF training, the L-BFGS algorithm is utilized for gradient descent optimization and getting model parameters. We apply randomized search using *RandomizedSearchCV* (https://scikit-learn.org/stable/modules/generated/sklearn.model_selection.RandomizedSearchCV.html) to find the best parameter values for *L1* (Lasso) and *L2* (Ridge) regularization coefficients in the CRF algorithm. The hyperparameter search is done by applying 5-fold cross-validation. In addition, we set the number of parameter settings to 20.

In the BLSTM-based experiments, we use a grid search algorithm for hyperparameter tuning. First, we set several values to find the best values for learning rate, batch size, dropout, and the number of LSTM units. The best learning rate and dropout value for all models are 0.01 and 0.5, respectively. The batch size and LSTM units for BLSTM and BLSTM + character CNN architectures are 32 and 64, respectively. As for BLSTM-CRF and BLSTM + character LSTM, the batch size and LSTM units are 64 and 32, respectively. Then, the BLSTM-based models are trained using the Adam optimizer over 20 epochs. Table 2 provides the best hyperparameter values for each BLSTM-based model.

**Table 2:** Best hyperparameter values for each BLSTM-based model.

**Model**	**Learning rate**	**Batch size**	**Dropout**	**LSTM units**
BLSTM	0.01	32	0.5	64
BLSTM-CRF	0.01	64	0.5	32
BLSTM + character LSTM	0.01	64	0.5	32
BLSTM + character CNN	0.01	32	0.5	64

We also employ a grid search algorithm for fine-tuning BERT models. The hyperparameter search is conducted by setting categorical values for learning rate, per device training batch size, and per device evaluation batch size. The learning rate is set to 1e−4, 3e−4, 2e−5, 3e−5, and 5e−5. For the per-device train and evaluation batch size, we select the categorical values of 8, 16, 32, 64, and 128. Subsequently, the hyperparameter search is run by applying such defined values over four epochs and ten trials. The best hyperparameter values for each BERT-based model are presented in Table 3. Finally, we use the best hyperparameters with the Adam optimizer and five epochs for fine-tuning BERT training.

**Table 3:** Best hyperparameter values for each BERT-based model.

**Model**	**Learning rate**	**Per device train batch size**	**Per device evaluation batch size**
mBERT	3e−05	64	32
IndoBERTweet	2e−05	32	8

## Results and Discussion

### Data annotation result

As described in Table 4, the dataset of this study consists of 11,019 tweets. We tokenize each tweet and annotate each token with the corresponding language label. As a result, our dataset comprises 273,323 tokens, 44,809 unique tokens, a tweet-length average of 24.8 tokens, and a token length average of 4.63 characters per token.

**Table 4:** Dataset information.

Number of tweets	11019
Number of tokens	273323
Number of unique tokens	44809
Average token length	4.63 characters
Average sentence length	24.8 tokens

The Indonesian language dominates our dataset with 144,934 tokens (53%). The number of OTH (Other) is 62,254 tokens (22.8%), followed by Javanese and English with 29,835 tokens (10.9%) and 28,058 (10.3%), respectively. For the intra-word code-mixing data, Indonesian-English has the highest frequency among the intra-word code-mixing labels with 5,625 tokens (2.1%). In contrast, the number of intra-word code-mixing labels for Indonesian-Javanese and Javanese-English are 1,557 (0.6%) and 1,060 (0.4%), respectively. Table 5 shows the dataset statistics used in our study.

**Table 5:** Dataset statistics.

**Label**	**Number of tokens**	**Percentage**
Indonesian (ID)	144,934	53%
Javanese (JV)	29,835	10.9%
English (EN)	28,058	10.3%
Mixed Indonesian-English (MIX_ID_EN)	5,625	2.1%
Mixed Indonesian-Javanese (MIX_ID_JV)	1,557	0.6%
Mixed Javanese-English (MIX_JV_EN)	1,060	0.4%
Other (OTH)	62,254	22.8%
Total	273,323	100%

We evaluate the annotation results by measuring inter-annotator agreement based on the annotation results using Cohen’s kappa. The agreement between the annotators attains a value of 0.9964. This result represents an almost perfect agreement between two annotators. As for the CMI, we gain a score of 38.05. It means that 38.05% of the overall non-neutral language tokens in the dataset are code-mixed.

### Language identification results

In this sub-section, we provide the code-mixed language identification results from our experiments. Table 6 presents the macro and weighted score results for precision, recall, and F1 for each model on the test set. In addition, Table 7 illustrates the comparison of the average F1 scores by category for the proposed models.

**Table 6:** Precision, recall, and F1 score results on the test set for each model.

**Model**	**Precision**		**Recall**		**F1 score**	
	**Macro**	**Weighted**	**Macro**	**Weighted**	**Macro**	**Weighted**
CRF	94.39	95.71	91.60	95.71	92.94	95.69
BLSTM	93.81	93.78	88.73	93.68	91.06	93.66
BLSTM-CRF	92.35	92.87	87.94	92.65	89.91	92.67
BLSTM + character LSTM	91.78	94.26	90.31	94.23	91	94.2
BLSTM + character CNN	93.51	93.69	89.04	93.67	91.11	93.66
mBERT	93.09	94.87	92.85	94.85	92.96	94.85
IndoBERTweet	93.21	95.64	93.86	95.63	93.53	95.63

**Table 7:** Category-wise comparison of F1 scores for each model.

**Label**	**CRF**	**BLSTM**	**BLSTM-CRF**	**BLSTM + character LSTM**	**BLSTM + character CNN**	**mBERT**	**IndoBERTweet**
EN	94.17	90.38	85.84	92.07	90.18	92.39	93.60
ID	97.11	95.45	95.05	95.94	95.58	96.50	97.06
JV	92.89	90.14	88.07	89.73	89.9	90.89	92.26
MIX_ID_EN	93.66	92	91.92	91.33	92.21	94.86	95.55
MIX_ID_JV	86.74	86.13	85.82	82.95	85.68	89.11	87.60
MIX_JV_EN	91.19	90.37	90.14	91.25	91.38	92.87	92.94
OTH	94.84	92.94	92.52	93.76	92.85	94.12	95.71

We infer that the CRF model performs well in determining languages from code-mixed texts. The CRF model achieves macro and weighted F1 scores of 92.94% and 95.69%, respectively. Also, the CRF shows good performance in identifying individual languages for non-intra-word code-mixing. Compared to the CRF model, there is no significant improvement in the BLSTM-based architectures. Furthermore, adding a CRF layer on top of BLSTM cannot improve the F1 score. However, combining word and character CNN embedding can slightly enhance the macro F1 score. Among the BLSTM-based architectures, the BLSTM and BLSTM + character CNN models indicate comparable results with macro F1 scores of 91.06% and 91.11%, respectively. In addition, both models achieve the same value for weighted F1 of 93.66%.

Our experiments with the fine-tuned BERT models indicate better results than the BLSTM-based models. The results provided by the fine-tuning BERT models demonstrate competitive performance compared to the other techniques. The fine-tuning IndoBERTweet model achieves the highest macro F1 score of 93.53% among the other models. This score is higher by 0.57% than the fine-tuning using mBERT, which gains a 92.96% F1 score. Further, the fine-tuning of BERT models proves an excellent achievement in identifying intra-word code-mixing.

### Error prediction analysis

This sub-section discusses the error prediction analysis generated by the models. We randomly pick a sample from the test set to be analyzed as follows:

***Tweet*:** *malah gua yg diblock, barusan bgt tb2 profilenya gabisa dibuka wkwkwk*

***English*:** *instead, I was blocked, just now suddenly the profile can’t be opened lol*

From the example above, the token *‘profilenya’* is mixed Indonesian-English (MIX_ID_EN). It consists of *‘profile’* (English) and the Indonesian suffix *‘-nya’*. Among all models, the fine-tuned IndoBERTweet model can correctly identify such a token as mixed Indonesian-English. However, the remaining models identify it as an Indonesian word. This misclassification happens due to the spelling similarity between Indonesian and English. The term *‘profile’* is written as *‘profil’* in Indonesian, while in English, it is written as *‘profile’*.

In addition, our models sometimes get confused when identifying between mixed Indonesian-English or Javanese-English. This confusion occurs since Indonesian and Javanese share similar morphemes. For example, the prefix *‘di-‘* in the following tokens, such as *‘didownload’* (downloaded), *‘dicancel’* (cancelled), *‘diclick’* (clicked), and *‘diprint’* (printed) can be identified as mixed Indonesian-English or mixed Javanese-English, depending on the sentence’s context.

Based on our investigation, the fine-tuning BERT and BLSTM-based models can generate more accurate language identification than the CRF. Both fine-tuning BERT and BLSTM-based models can grasp the meaning of each word depending on the context. Also, we do not need to define the features manually for fine-tuning and BLSTM-based models. However, the fine-tuning BERT models can produce better identification than the BLSTM-based models. Unlike directional models, which read a text from either left-to-right or right-to-left, the BERT models can read a text or a sequence of words simultaneously in any direction. Hence, the BERT model can understand the meaning of the given sequence based on the context to its right and left.

On the contrary, relying on CRF’s handcrafted features is unreliable, especially in determining intra-word code-mixing. For example, the CRF model fails to identify words that have suffix similarity with Javanese, such as *’-re’, ’-ke’,* and *’-ne’*. As a result, some words like *’atmosphere’, ’Singapore’, ’alike’,* and *’airplane’* are identified as mixed Indonesian-Javanese (MIX_ID_JV) by the CRF model. This is due to the fact that such words were not encountered during the CRF training.

Furthermore, we observe that tokens with JV labels are incorrectly detected as ID occasionally. This misclassification happens because of the shared vocabularies between Indonesian (ID) and Javanese (JV), such as *‘aku’* (I)*, ‘bareng’* (together), *‘kuat’* (strong), and *‘buku’* (book). Our models sometimes incorrectly predict the English (EN) tokens as Indonesian (ID). Misclassification from EN to ID mainly occurs when Indonesian words surround an English word. Words recognized in Indonesian and English sometimes confuse the model to predict the correct label, for instance, *‘internet’*, *‘admin’*, *‘hotel’*, and *‘got’.* Apart from that, some English words that are written in Indonesian or abbreviated styles also make our model confused to predict the correct label, like *‘kambek’* (come back), *‘mensen’* (mention), *‘gaes’* (guys), *‘fllw’* (follow), and *‘pls’* (please). The actual label for such words is English, but those words are predicted as Indonesian.

## Conclusion and Future Work

In this article, we developed a code-mixed Indonesian-Javanese-English corpus for language identification (IJELID) using Twitter data. To standardize the annotation results, we created an annotation guideline for annotators. Also, we discussed some challenges encountered during corpus creation. To build the code-mixed language identification models, we explored various techniques, such as fine-tuning BERT, BLSTM-based, and CRF. Our experiments indicate that the fine-tuned IndoBERTweet models can effectively identify languages from the code-mixed tweets. Furthermore, using sub-word language representation in BERT models can produce a reliable model to identify languages for code-mixed texts. In addition, the BERT models can infer the context of each word from the given sequence better than the other techniques.

Even though the results of this research using BERT have demonstrated excellent performance, some spaces can still be improved from our study. In this study, we performed fine-tuning on pre-trained multilingual and monolingual models. Therefore, we intend to build a pre-trained model from code-mixed texts. We aim to investigate the impact of utilizing a pre-trained code-mixed model compared to the pre-trained monolingual and multilingual models.

## Additional Information and Declarations

### Data Availability

The following information was supplied regarding data availability: The data is available at Zenodo: Hidayatullah, Ahmad Fathan. (2022). Code-mixed Indonesian-Javanese-English Twitter Dataset (Version v1) [Data set]. Zenodo. https://doi.org/10.5281/zenodo.7567573.
